# Heart Failure from Over-Strain

**Published:** 1901-07-06

**Authors:** 


### Hospital Clinics and Medical Progress.

### HEART FAILURE FROM OVER-STRAIN.

In delivering the Cavendish Lecture on "Acute Cardiac Failure," Sir Richard Douglas Powell commenced by emphasising a point which we think is far too often forgotten by those who talk learnedly on heart disease—namely, that the heart is but a part, differentiated for special reasons of a complete cardio-vascular tubal system containing the blood. The tubes are no passive tubes sluiced by a central force pump. On the arterial side the powerful rhythmic pulsations of the heart are so modified and restrained by the elastic and muscular action of the vessels as to ensure a safe and adequate stream of blood throughout the body; whilst on the venous side the position of the valves, the action of the adjacent and often ensheathing muscles, the impetus of the lymph and chyliferous tributary currents, and the rhythmically-acting thoracic aspiration, help to maintain the blood movement. Obviously enough a circulation which is dependent on influences so widely spread demands also the co-ordinating power of a widely-distributed nervous system by which it is brought in touch with many disturbing forces, while one must not omit the condition of the blood itself as influencing the facility with which the circulation can take place. With such an infinite number of contributory factors influencing the circulation, cardiac failure can never be a simple problem. But taking the comparatively simple example in which acute heart failure occurs in a young and healthy heart from overtaxation, Sir Douglas Powell says that there are in such cases always two factors at work, namely, direct fatigue of the neuro-muscular tissue of the heart, and a poisoning of the blood, and thus of the nerves and muscles, of auto-metabolic source. As showing how largely the toxic element is concerned in the process it is pointed out that, amongst the concomitants of heart distress coming on during violent exercise, such as running, vomiting is one of the most common. A boy will say that at the end of a long race which overtaxed him he cast himself on the ground and vomited. Sometimes the boy will finish his run merely fagged, or he will faint or vomit by the way, and have to lie down in the road. Or he may finish his run, but vomit all the evening. All this points to poisoning. But the strain may have still more serious effects. He may become absolutely insensible while running and remain so for hours, or he may even die on the road. There is much reason to believe that such heart failure is largely due to poisoning by the accumulated products of metabolism, and the vomiting so commonly met with in such cases is one proof of this. Another is the anæmia which is so often left as a consequence of such overstrain, showing as it does the changed condition of the blood which has resulted from the accumulation of the products of metabolism. The gastro-intestinal attacks—vomiting or diarrhœa—which commonly occur when those who habitually lead a sedentary life suddenly take to exhausting exercises, point in the same direction, and even more certain proofs of altered blood are to be found in the bodies of those who have died from excessive exercise, and in hunted animals, of which increased liquidness of the blood, absence of post-mortem clotting and darkened colour are among the more obvious. "We are all of us familiar," says Sir Douglas Powell, "with cases of neurasthenia and anæmia distinctly traceable to some severe exertion in a person with no previous training." In consequence of the fact that the heart of the child, between six and twelve years of age, is naturally relatively hypertrophied, a slight extension of the apex beat within the nipple line, unaccompanied with confirmatory signs, need not be taken as a necessarily morbid condition when met with in such a child. One must look for evidence of overstrain rather in an extension of the upper margin of dulness in the midsterno nipple line, an unduly perceptible impulse at the ensiform base, and in hesitating action of the heart—*i.e.* an irregularity in rhythm and force. These signs in association with languor, anæmia, breathlessness on exertion, and sometimes a sharp pain at the heart which a boy may describe as "a beastly pain catching the wind" caused by a slight effort, are sufficient for diagnosis. There may be other signs, such as murmurs, but murmurs are not essential. A cardinal point to be borne in mind with regard to young boys and girls is their special aptitude for short spells of active exercise, but their complete unfitness for prolonged fatiguing monotonous exertion. This is almost the reverse of what is the case in older people, and is very apt to be forgotten, not perhaps so much at school as at home. "Athletic fathers, robust elder brothers, proud of the sporting alertness of young boys, will often keep them out on long bicycle, golf, or shooting expeditions, and may thus cause grave damage to the young and rapidly developing heart." The treatment of acute heart failure from overstrain involves a few weeks' complete rest and often many months of careful supervision. Young people make a rapid and generally a complete recovery, provided no actual lesion has been produced, and the younger they are the more ready and complete is the recovery likely to be, but in youths and young men a degree of irritability of heart is often to be observed for many months, even for some years, the patient suffering from attacks of palpitation and cardiac pain on slight fatigue or exertion, and often at night. Quiet exercise should be resumed in such cases after a time, and it is better to allow interesting exercises within measure, such as croquet, level cycling, golf, and easy tennis, rather than more formal courses of training.

